# The Hepatocyte Growth Factor (HGF)/Met Axis: A Neglected Target in the Treatment of Chronic Myeloproliferative Neoplasms?

**DOI:** 10.3390/cancers6031631

**Published:** 2014-08-12

**Authors:** Marjorie Boissinot, Mathias Vilaine, Sylvie Hermouet

**Affiliations:** 1 Translational Neuro-Oncology Group, Leeds Institute of Cancer and Pathology, University of Leeds, Level 5 Wellcome Trust Brenner Building, St James’s Hospital, Leeds LS9 7TF, UK; E-Mail: m.boissinot@leeds.ac.uk; 2 Institute of Research on Cancer and Aging (IRCAN), CNRS-Inserm-UNS UMR 7284, U 1081, Centre A. Lacassagne, 33 Avenue Valombrose, Nice 06189, France; E-Mail: mathias.vilaine@unice.fr; 3 Centre Hospitalier Universitaire (CHU), Place Alexis Ricordeau, Nantes 44093, France; 4 Inserm UMR892, Centre de Recherche en Cancérologie Nantes-Angers, Institut de Recherche en Santé, Université de Nantes, 8 quai Moncousu, Nantes cedex 44007, France

**Keywords:** MET, hepatocyte growth factor (HGF), myeloproliferative neoplasm (MPN), chronic myelogenous leukaemia (CML), MET expression, HGF blood levels, dependence factor, personalised medicine, MET inhibitors, HIF inhibitors

## Abstract

Met is the receptor of hepatocyte growth factor (HGF), a cytoprotective cytokine. Disturbing the equilibrium between Met and its ligand may lead to inappropriate cell survival, accumulation of genetic abnormalities and eventually, malignancy. Abnormal activation of the HGF/Met axis is established in solid tumours and in chronic haematological malignancies, including myeloma, acute myeloid leukaemia, chronic myelogenous leukaemia (CML), and myeloproliferative neoplasms (MPNs). The molecular mechanisms potentially responsible for the abnormal activation of HGF/Met pathways are described and discussed. Importantly, inCML and in MPNs, the production of HGF is independent of Bcr-Abl and *JAK2*V617F, the main molecular markers of these diseases. *In vitro* studies showed that blocking HGF/Met function with neutralizing antibodies or Met inhibitors significantly impairs the growth of *JAK2*V617F-mutated cells. With personalised medicine and curative treatment in view, blocking activation of HGF/Met could be a useful addition in the treatment of CML and MPNs for those patients with high HGF/MET expression not controlled by current treatments (Bcr-Abl inhibitors in CML; phlebotomy, hydroxurea, JAK inhibitors in MPNs).

## 1. Introduction

The mounting evidence that alteration of the same genes (*IDH1/2*, *EZH2*, *TET2*, *MLL*…) can be found in solid tumours and in haematological malignancies highlights the similarity of oncogenic mechanisms in human malignancy, independently of the tissue origin of the cancer stem cells [1,2,3,4,5,6]. For certain genes however, the knowledge accumulated in solid cancer studies translates poorly to blood malignancies: the *MET* gene, which encodes the tyrosine-kinase receptor for hepatocyte growth factor (HGF), is one example. *MET* mutation and amplification are well describedin solid tumours, and the*MET* product (Met) is the target of numerous clinical trials aiming to personalise treatment for various types of cancers [7,8,9,10,11]. In contrast, studies of the *MET* gene in haematological malignancies are relatively few. This is quite surprising because the blood serum levels of HGF have been reported to be abnormal in myeloma, acute myeloid leukaemia (AML), chronic myelogenous leukaemia (CML) andmyeloproliferative neoplasms (MPNs) [12,13,14,15]. Importantly, malignant CML and MPN progenitors produce HGF in an autocrine fashion, and HGF expression levels were reported to have significant prognosis impact in AML and in CML [15,16,17].

**Figure 1:**
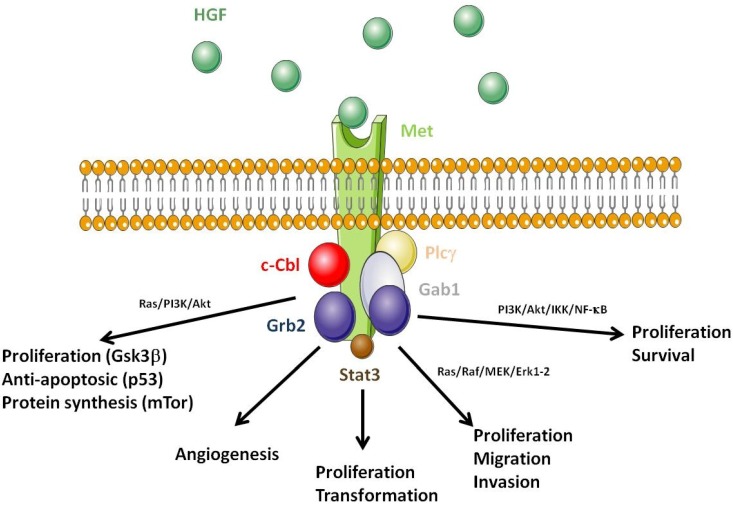
Consequences of the disruption of hepatocyte growth factor (HGF)/Met function. HGF (green circles) binding to Met induces receptor dimerization; the Met kinase is then activated by auto-phosphorylation of tyrosine residues. Met activation induces the recruitment of proteins acting as adaptors for other downstream signalling partners. Amongst them are: Grb2, the PI3K p85 subunit, PLCγ, Stat3, and Gab1. Met activation generates signalling pathways involved in proliferation, migration and invasion via Ras/Raf/MEK/Erk1/2; proliferation and transformation via Stat3; angiogenesis, proliferation and survival via PI3K/Akt/IKK/NF-κB; and proliferation-anti-apoptotic effect-protein synthesis via PI3K/Akt-Gsk3β-p53-mTor. The U3 ubiquitin ligase c-Cbl is also recruited to Met after activation and is responsible for Met ubiquitinylation and targeted degradation by the proteasome.

Both the *HGF* and *MET* genes are located on chromosome 7, a chromosome frequently altered in haematological malignancies. HGF is produced as a one-chain inactive pro-protein, later cleaved into a two chain (α, β) biologically active form by enzymes such as HGF activator (HGFA). Other enzymes such as thrombin, type II transmembrane enzyme matriptase, hepsin and uPAR also cleave pro-HGF into HGF [18]. Met is formed by a 50 kDa α sub-unit linked by a disulphide bond to a 145 kDa β chain. Upon ligand binding and subsequent dimerization, the β chain carries the signal transduction via auto-phosphorylation of its tyrosine kinase domain. Met autophosphorylation at residues Y1234/Y1235 in the activation loop of the kinase domain provide loop movement and full catalytic activity. Phosphorylation sites in the carboxy-terminal region (Y1249 and Y1256) are required for docking, as well as for biological activity. Phosphorylated Met recruits several signalling molecules including the growth factor receptor-bound protein 2 (Grb2), Shc, the p85 subunit of phosphatidylinositol 3' kinase (PI3K), the phospholipase C γ (PLCγ), the signal transducer and activator of transcription 3 (Stat3) and the Grb2-associated binding protein 1 (Gab1) (Figure 1) [19,20,21,22,23,24]. Met activation provides signalling for migration via Ras/Raf/MEK/Erk1/2; for cell proliferation and transformation via Stat3; for angiogenesis, proliferation and survival via PI3K/Akt/IKK/NF-κB; and anti-apoptotic effect and protein synthesis via PI3K/Akt, Gsk3β, p53 and mTOR [25,26,27,28,29,30]. After Met activation, the U3 ubiquitin ligase c-Cbl is recruited to Met, to ubiquitinilate the receptor in view of its degradation by the proteasome [31]. Under stress conditions, Met is cleaved by a caspase-3-mediated mechanism that generates a 40 kDa fragment responsible for the induction of cell apoptosis [32]. Met also interacts with integrins, CD44/heparin and class B plexins via HGF-dependent and independent mechanisms [33]. Thus, an additional potential role for HGF/Met in haematopoiesis is the mobilisation of progenitor cells in synergy with G-CSF [34]. Like G-CSF, HGF induces matrix metalloproteinase 9 (MMP-9), which facilitates cell mobilization from the bone marrow to the peripheral blood.

The molecular mechanisms for increased HGF expression in malignant myeloid cells are not known. Thus, a better understanding of the role played by HGF in human haematological malignancies is necessary, and important for several reasons: HGF is a multifunctional, pleiotropic, pro-survival cytokine, which stimulates early myelopoiesis, is strongly anti-inflammatory, andproduced by many tumoural cells [16,17,35,36,37,38,39,40,41,42,43]. Consequently, diverse new molecules aiming to block the HGF/Met axis are now being tested in clinical trials [7,8,9]. These new therapeutic options should be of interest in myeloid malignancies, notably MPNs, a group of diseases withchronic inflammation wherevery high HGF levels are frequent [44,45,46,47,48,49,50]. The object of this review is to gather and summarise published studies of the *MET* gene in myeloid malignancies, and to provide evidence that drugs currently used in solid tumours to block the HGF/Met axis should also be considered for the therapy of chronic myeloid malignancies.

## 2. Chronic Myeloproliferative Neoplasms

**Figure 2:**
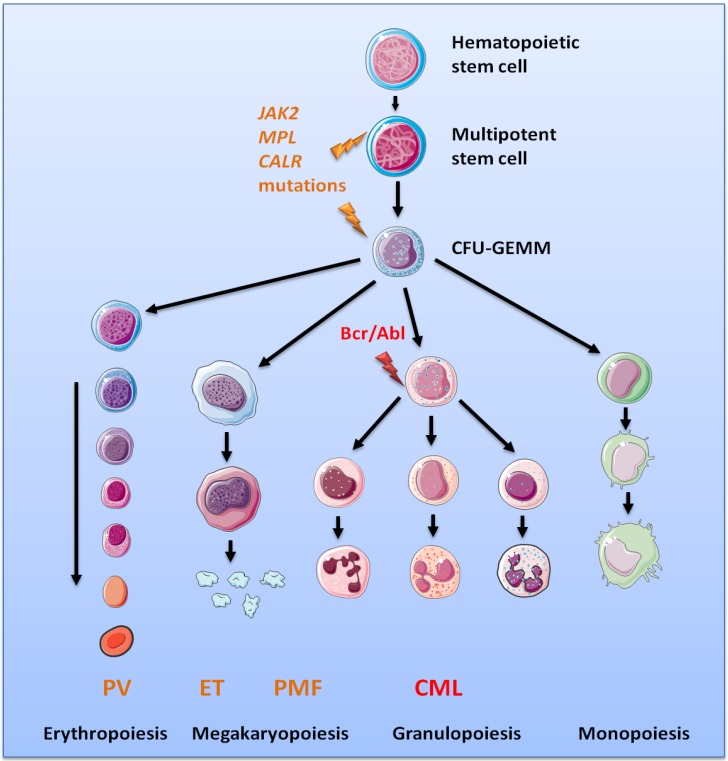
Chronic myeloproliferative neoplasms and main biological markers. Normal haematopoiesis takes place in the bone marrow where a haematopoietic stem cell, under cytokine stimulation in combination with cell-cell contacts progresses to multipotent stem cell state and then to Colony-Forming-Unit of Granulocyte-Erythrocyte-Megakaryocyte-Monocyte (CFU-GEMM) progenitor. This CFU-GEMM progenitor is capable of producing cells of all myeloid lineages following an array of differentiation processes which rely on cytokine stimulation: Erythropoietin (Epo) for erythropoiesis; Tpo for megakaryocytopoiesis; granulocyte-colony stimulating factor (G-CSF) for the production neutrophils; and GM-CSF for the production of granulocytes, monocytes and macrophages. In CML, the t(9;22) (q34;q11) translocation results in the Bcr-Abl fusion protein responsible of the abnormal proliferation of the granulocytic lineage. MPNs are characterised by mutations in the *JAK2*, *MPL* or *CALR* genes in a multipotent stem cell or CFU-GEMM. Mutations in the *JAK2* gene are implicated in the transduction of Epo, Tpo and G-CSF, whereas mutations in the *MPL* gene affect mostly the transduction of Tpo. These mutations result in abnormal erythropoiesis or megakaryocytopoiesis, often associated with elevated granulocyte counts. Regarding *CALR* mutations, their impact on the quantity and function of signalling molecules in myeloid cells still needs to be elucidated.

Chronic myeloproliferative neoplasms are a family of rare hematologic diseases that include CML, MPNs, chronic eosinophilic leukaemia, mastocytosis, and unclassifiable MPNs [51]. This review focuses on CML and MPNs. Chronic myeloproliferative neoplasms are characterized by the clonal proliferation of one or several myeloid lineages, associated in some cases with bone marrow fibrosis, splenomegaly and/or hepatomegaly. The two major diseases, CML and MPNs, are classified based on the presence or the absence of the *BCR-ABL* fusion gene that is the hallmark of CML [52]. Regarding MPNs, three subtypes are recognized: polycythaemia vera (PV), essential thrombocythemia (ET) and primary myelofibrosis (PMF). Three different types of molecular markers are known in MPNs: activating mutations in the *JAK2*gene (*JAK2*V617F being the most frequent mutation, found in all subtypes of MPNs); activating mutations in the*MPL* gene (*MPL*W515L/K mostly, detected only in ET and PMF); and diverse alterations of the gene encoding calreticulin (*CALR*), also typical of ET and PMF (Figure 2, Table 1) [53,54,55,56,57,58,59,60,61]. A small percentage of MPN patients do not carry any of the above mutations. *MPL* encodes the receptor for thrombopoietin (Tpo), which is coupled to the tyrosine kinase Jak2. Activation of the Mpl/Jak2 pair results in activation of the Jak2/Stat5 pathways, critical for myelopoiesis. Although some *CALR* mutants were also reported to lead to Stat5 activation, the mechanisms of action of *CALR* mutants remain unclear.

CML is an excessive, chronic proliferation of clonal granulocytic progenitors, associated with extramedullary haematopoiesis, and splenomegaly [52,62]. The *BCR-ABL*fusion gene results from the t(9;22) (q34;q11) translocation, the so-called Philadelphia chromosome [52]. The*BCR/ABL* fusion gene produces p210 Bcr-Abl, a constitutively active kinase; CML patients may present Bcr-Abl transcript variants. CML abnormal production of granulocytes is due to the Bcr-Abl fusion proteins, which inducecytokine-independent cell proliferation via the stimulation of signalling pathways crucial for myeloid cellsurvival and proliferation, including Ras/Raf/MEK/Erk1/2, PI3K/Akt, Stat5/Bcl-xl and NF-κB [63,64,65,66,67,68]. Acquisition of secondary genetic defects over time leads to disease progression from chronic phase to accelerated phase and blast crisis, with increased risk of death for patients. Twenty years ago, the median survival for CML patients was less than six years but thanks to the Bcr-Abl inhibitor imatinib (Gleevec^®^), the prognosis of CML has changed radically [69]. Imatinib was the first drug to offer the possibility to achieve molecular remission in CML. Unfortunately, imatinib does not cure CML, and resistance is not rare.

**Table 1:** Main characteristics of chronic myeloproliferative neoplasms.

		MPNs		
	CML	ET	PV	PMF
**Main phenotype**	Granulocytosis Basophilia	Thrombocytosis	Polycythaemia	Abnormal megakaryocytopoiesis Variable blood counts
**Main genetic alteration(s)**	Bcr-Abl rearrangement	*JAK2*V617F (heterozygous) *CALR* mutants *MPL*W515L/K mutants	*JAK2*V617F (homozygous)	*JAK2*V617F (heterozygous or homozygous) *CALR* mutants *MPL*W515L/K mutants
**Main activated pathway**	Abl	Jak2/Stat5 Jak2/Stat1 Protein trafficking (*CALR* mutants)	Jak2/Stat5	Jak2/Stat5 Protein trafficking (*CALR* mutants)
**Inflammation**		Mild	Variable	Severe
**Myelofibrosis**	Variable	Limited	Variable	Severe
**Hepato-splenomegaly**	Variable /Severe	Rare	Variable	Severe
**HGF levels**	High	Moderately elevated	High	High
**Met levels **	Absent in chronic phase; present in acute phase	Not studied	High in erythroblasts	Not studied
**Main treatment**	Bcr-Abl inhibitors (imatinib…)	Salicylic acid Hydroxyurea	Phlebotomy Hydroxyurea Interferon-α	Hydroxyurea Interferon-α Jak inhibitors
Clinical and molecular response * (main disease marker)	Yes	No	Only with Interferon-α2a	No
Cure*	No	No	No	No

* With current treatments.

MPNs are a heterogeneous group composed of three distinct diseases, ET, PV and PMF (Table 1). ET is characterized by megakaryocytic hyperplasia and platelet counts above 450.10^9^/L; patients may present with elevated granulocyte counts and splenomegaly. PV is a clonal expansion of erythroid progenitors, often associated with leukocytosis and thrombocytosis [51,70,71,72,73,74]. ET and PV are relatively indolent, whereasPMF is an aggressive disease characterised by high proliferation of myeloid cells, abnormal megakaryopoiesis, fibroblast proliferation and reticulin and collagen release leading to bone marrow fibrosis, extramedullary haematopoiesis, and splenomegaly. Mutations have been detected in MPNs in three genes: *JAK2*, *MPL*, and *CALR*. The activating *JAK2*V617F mutation is the most frequent: It is present in 95% of PV, 70% of ET, and 50% of PMF [53,54,60]. *JAK2*V617F leads to increased activity ofthe Jak2/Stat5 pathways, the tyrosine kinase which signals downstream of the receptors for Epo, Tpo and G-CSF, critical cytokinesfor myelopoiesis. *MPL*W515L/Kare also activating mutations; like *JAK2*V617F, they result in the activation of the Jak2/Stat5 pathways, whereasmutant forms of calreticulin, a protein chaperone, may alter protein trafficking. It is not fully understood why the same *JAK2*, *MPL* or *CALR* mutations are found in very different MPN subtypes.

Regarding therapy, PV patients are treated with phlebotomy and hydroxyurea (HU); ET patients receive salicylic acid or HU; and PMF patients are treated with HU, interferon-α (IFN-α) or allogenic bone marrow graft. Following the discovery of the *JAK2*V617F mutation, several JAK inhibitors, including ruxolitinib, were developed. PMF patients were the first MPN patients to test ruxolitinib: despite significant regression of secondary symptoms of PMF disease linked to inflammation such as splenomegaly, fever, night sweats or weight loss, ruxolitinib had little or no impact on the *JAK2*V617F clone [75,76]. Ruxolitinib has also been tested in a murine model of PV and in patients with advanced PV, again with reduction of secondary symptoms only [77,78]. Currently, no drugs exist that target Mpl or calreticulin mutants. Logically, since Jak1 transmits the signalling of several major inflammatory cytokines, the Jak inhibitors that proved most efficient on spleen size and other inflammation-linked symptoms in MPNs are those which inhibit Jak1 in addition to Jak2. This suggests that targeting inflammation as well as the Jak2, calreticulin or Mpl mutants is important in the treatment of MPNs.

In contrast, the use of imatinib mesylate (Gleevec^®^) for the treatment of CML is the biggest success in the development of specific tyrosine kinase inhibitors (TKIs). Perhaps one reason is that in addition to Bcr-Abl, imatinib targets the ATP binding site of other tyrosine kinases important for myeloproliferation and for fibrosis, notably c-Kit, the receptor for stem cell factor (SCF), and the receptor for platelet-derived growth factor (PDGF). SCF and PDGF are important factors for the survival and growth of myeloid cells and fibroblasts, respectively. With imatinib treatment, more than 80% of CML patients achievepartial or complete molecular response, resulting in remission or extended survival in chronic phase of CML. Recently Mahon *et al.* reported that long term arrest of treatment was possible without relapse [79]. However CML patients risk toxicity and drug-resistance, and despite second (Dasatinib, Nilotinib) and third (Ponatinib) generation TKIs, resistance does occur due to mutations or amplification of the *BCR-ABL* fusion gene, or to various Bcr/Abl-independent resistance mechanisms, such as mutations in genes encoding other tyrosine kinases (Src-kinase related *LYN*, *KIT*, *MET*) or deregulation of the expression of drug influx protein Oct-1 [62,80,81,82,83,84]. Thus, curing CML with TKIs alone is unlikely, and various combination treatments are being tested. Zhang *et al.* developed a strategy based on imatinib and pan-histone deacetylase (HDAC) inhibitors, which induced a significant reduction of quiescent CML stem cells but unfortunately had a high toxicity on normal hematopoietic stem cells [85]. Recent studies demonstrated the relative efficacy on CML cells of imatinib in combination with Jak2 inhibitor TG101348 (cytostatic effect), the CXCR4 antagonist BKT140, or the proteasome inhibitor carfilzomib (cytotoxic effect but 50% of cell death only) [86,87,88]. Previously acombination of imatinib with pegylated IFN-α2b had shown a high rate of molecular response in low or intermediate chronic phase CML [89].

## 3. HGF and Metexpression in CML and in MPNs

HGF is originally produced as an inactive form, pro-HGF, which is stored in the matrix and later cleaved into active HGF. The stromal matrix also acts as a reservoir for HGF, due to its high affinity for heparin sulphate proteoglycans and to a lesser extent, to thrombospondin-1, fibronectin, laminin, collagen type I, and basement membranes. Met, the receptor tyrosine kinase specific to HGF, is expressed bybone marrow fibroblasts and CD34+ hematopoietic progenitors, including BFU-E and CFU-GM.

### 3.1. Paracrine and Autocrine HGF Production and Autocrine HGF/Met Loop

**Figure 3:**
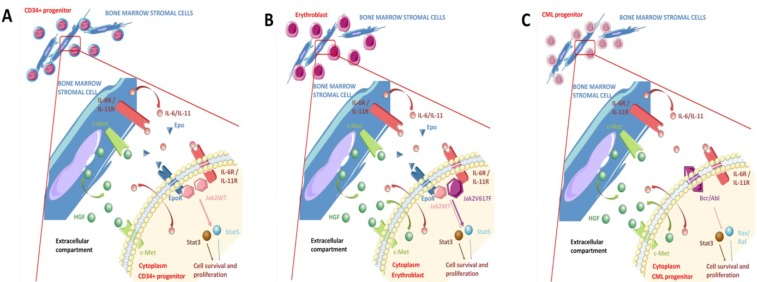
Paracrine and autocrine HGF production and autocrine HGF/Met loop in myeloproliferative neoplasms (MPN) and chronic myelogenous leukaemia (CML) progenitors. (**A**) In physiological conditions, CD34+ haematopoietic progenitors secrete little or no HGF and bone marrow stromal cells (BMSCs) are the main source of HGF. HGF stimulation and activation of Met in myeloid progenitors is therefore mostly paracrine; (**B**) In MPNs, both non-mutated BMSCs and clonal, *JAK2*V617F-mutated progenitors produce HGF. Thus, activation of the HGF/Met axis in MPN progenitor cells is autocrine and paracrine. In polycythaemia vera (PV) erythroid progenitors, HGF production is independent from *JAK2*V617F; (**C**) Similarly, in CML both non-mutated BMSCs and Bcr-Abl-rearranged basophils produce HGF, and activation of the HGF/Met axis is autocrine and paracrine. HGF production is independent from Bcr-Abl.

Normal haematopoietic progenitors produce little or no HGF, whereas bone marrow stromal cells (BMSCs) are a physiological source of HGF. Thus, in physiological conditions, the action of HGF on haematopoietic progenitors is mostly paracrine (Figure 3A) [90,91]. Neutralising anti-HGF antibodies block the ability of BMSCs to promote colony formation by normal CD34+ progenitors, and decrease BMSC proliferation and adhesion to fibronectin and type IV collagen [18,90,91].

The deregulation of HGF production in myeloid malignancies is not a recent observation. In 1996, Hino *et al.* found increased levels of HGF in the bone marrow plasma of AML and CML patients, compared to normal controls and patients with acute lymphoid leukaemia (ALL) [92]. These findings have been confirmed by several groups, and extended to several other haematological malignancies. In particular, CD34+ progenitors from patients with AML, myelodysplatic syndrome (MDS) and CML were shown to express Met at their surface andto produce HGF, thus indicating an autocrine loop of activation of the HGF/Met pathway [18,92,93]. Similarly, HGF levels were also reported to be high in myeloma, both in the blood of patients and in supernatants from malignant plasma cells [94]. Moreover, plasma cells expressed HGF and Met, confirming an autocrine HGF/Met loop also in myeloma. HGF was shown to promote colony formation and migration of AML and MDS progenitor cells *in vitro* in a dose dependent manner.

In the following years, several groups confirmed the importance of the deregulation of HGF inmyeloid malignancies [95,96,97,98,99,100,101]. Verstovsek *et al.* reported increased levels of HGF in newly diagnosed AML and MDS patients *vs.* healthy controls, and correlated HGF levels with white blood cell counts [96]. Myeloblasts expanded from CD34+ bone marrow progenitors of CML patients were shown to secreteten times more HGF, VEGF, FGF-2 and IL-8 compared to healthy donors [97]. Mahadevan *et al.* published a case of mixed MDS/MPN later transformed into AML type M2; the patient had elevated HGF in serum but normal karyotype throughout the course of her disease [98]. Our group found HGF overexpressed in the serum, bone marrow plasma, and BMSC supernatants from PV patients compared to secondary erythrocytosis (SE) controls; we determined that BMSCs and clonal erythroblasts were the major sources of HGF in PV (Figure 3B) [15]. Using anti-HGF and anti-Met neutralising antibodies *in vitro*, we were able to inhibit the growth of *JAK2*V617F-mutated PV erythroblasts; similar inhibition was also observed in the *JAK2*V617F^+/+^ HEL cell line. Furthermore, serum levels of HGF from PV patients without *JAK2* mutation were as high as those of *JAK2*V617F-positive patients. We also demonstrated that induction in cell lines of *JAK2*V617F, or its knocking-down, did not affect HGF production, which indicated that the production of HGF by PV progenitors was not *JAK2*V617F-dependent [15]. Subsequent studies analysedthe levels of 30 cytokines in the plasma of PV and PMF patients: HGF was increased in both PV and PMF, compared to healthy donors, and HGF was correlated with leukocyte countsin PV and PMF, and also with splenomegaly in PMF [99,100]. Recently, HGF levels were also found to be elevated in ET patients [101].

In CML a high production of HGF was found associated with increased basophilia, a key feature of the acceleration phase in CML, and thus, HGF level was considered a highly significant prognostic factor [16]. For CML patients with HGF in serum and bone marrow, the level of HGF was correlated with the microvessel density in the bone marrow. The proportion of CML primary cells expressing HGF at the mRNA and protein levels was greater in patients in accelerated compared to patients in chronic phase. Basophils were identified as the main cellular source of HGF (Figure 3C) [16]. In addition, HGF production was confirmed in the basophil-committed cell line KU812, which also expresses Met. This highlighted a so far unknown active role for basophils in CML disease acceleration. Importantly, HGF expression levels were not affected by Bcr-Abl inhibitor imatinib, hereby establishing that in CML, HGF production is essentially independent from Bcr-Abl [16].

Thus, inflammation-linked HGFis over-expressedin MPNs and in CML, by BMSCs and by the malignant myeloid cells themselves, and HGF promotes colony formation and migration of AML and MDS progenitor cells *in vitro* in a dose-dependent manner. In malignant myeloid cells, HGF production was demonstrated to bemostly or totally independent from the two major biomarkers of these diseases, Bcr-Abl and *JAK2*V617F, respectively. Hence, HGF and its receptor Met could represent promising new targets to add to the current treatments for CML and MPNs.

### 3.2. Met as a Pro-Survival Receptor

So-called “dependence receptors” induce a specific death signal when unbound by their ligand. As a result, the survival of the cell becomes dependent on the presence of the ligand in the cellular environment. In physiological conditions, a controlled production of ligand will maintain the balance between survival and apoptosis and regulate cell numbers. In a malignant niche, overexpression of the ligand or chronic activation of the receptor will lead to increased cell survival [36]. As described above, there is ample evidence that malignant cells rely on the HGF/Met axis for survival, and that activation of the HGF/Met axis attenuates activated stress pathways andhas strong cytoprotective effects. Yet *MET* deletion or inhibition by neutralizing anti-HGF or anti-Met antibodies does not systematically lead to cell death; thus, Met is best considered as a pro-survival receptor rather than as a *bona fide* dependence factor. Here we give an overview of the mechanisms of activation and signalling related to Met function as cytoprotective, pro-survival factor.

Disruptionby over-expression of ligand orincreased receptor expression or function (mimicking ligand induced activation) leads to inappropriate survival and proliferation, thus favouring accumulation of genetic abnormalities and cell transformation. Activation of the HGF/Met axis also promotes cell invasiness, metastases, angiogenesis, and the production of inflammatory cytokines (Figure 3). In addition, HGF produced by BMSCs was recently shown to induce the expansion of CD14− CD11b+ CD33+ myeloid-derived suppressor cells (MDSCs) [102]. MDSCs are involved in immune tolerance, as they concomitantly inhibit the proliferation of CD4+ and CD8+ T cells and increase the production of regulatory T cells. By decreasing the immune response, MDSCs prevent malignant cells from being detected and destroyed. Thus, HGF secreted by AML blasts, CML basophils and MPN progenitors, could lead to the expansion of immunosuppressive MDSCs. Similar effects of HGF on MDSCs may be at work in patients with advanced stage solid tumour cancers. In such patients, plasma HGF levels are frequently significantly higher than normal, because many sources of disease-associated stress induce HGF production by the host.

Activation of the HGF/Met axis via *MET* mutation is frequent in cancer. *MET* mutations are detected in solid tumours such as hereditary papillary type I renal cell carcinoma, hepatocellular carcinoma, head and neck carcinoma, breast, gastric and ovarian cancers, and non-small cell lung cancer [103]. In CML and in MPNs, MET expression varies and no *MET* mutation has been reported, but HGF is very frequently overexpressed. In solid tumours, MET is over-expressed via transcriptional regulation through HIF-1α and deregulation of other transcription factors (AP-1, Sp1, Ets) or down-regulation of microRNAs (miR-1, miR-34, miR-449a) [104,105,106,107,108,109]. *In vitro* over-expression of wild type MET in primary human osteoblasts was sufficient to induce malignant transformation if a certain threshold expression was reached [110]. In CLL, BMSCs produce HGF that promotes CLL cell survival via the activation of the Met/Stat3 pathways; this effect could be blocked with the SU11274 MET inhibitor [111]. Logically, knock-out of *MET* sensitizes cells to cytokine-mediated cell death, whereas HGF protects cells from cell death, via inactivation of NF-κB [112].

### 3.3. Cross-Talk between the HGF/Met Axis and Cytokines Linked to Inflammation

HGF induces the production by BMSCs of interleukin (IL)-11, IL-10, IL-6, IL-8, stromal cell-derived factor 1α (SDF-1α), vascular endothelial growth factor (VEGF) and to a lesser extent SCF, in a dose dependent manner, whereas forced production of HGF reduces expression of IFN-γ, TGF-β, and TNF-α (Table 2) [113,114]. Inversely, anti-HGF antibodies lead to decreased mRNA levels of IL-11, SDF-1α and SCF. Interestingly, IL-11 and IL-6 promote myelopoiesis, especially the production of red blood cells (IL-11) and platelets (IL-6 and IL-11), and HGF and SCF act in synergy to promote the formation of BFU-E. SDF-1α plays an essential role in homing, repopulation and maintenance of stem cells and in synergy with SCF, contributes to the maintenance of myeloid progenitors. Thus, signalling triggered downstream of HGF/Met modulates the production of cytokines known to regulate multiple cellular processes: angiogenesis, proliferation, migration and survival. This highlights the paramount role of HGF/Met regulation in maintaining a normal haematopoietic niche, and the extent of the damage potentially caused by deregulation of the HGF/Met axis, notably in promoting and maintaining malignancy.

**Table 2:** Regulation of *HGF*/*Met* expression, and cytokines regulated by HGF/Met.

Regulators of HGF and Met expression		
	**HGF**	**Met**
**Stimulants**	b-FGF, IL-3, OSM IFN-γ (only weakly) HIF-1α NF-κB	SCF IL-3 IL-11
**Inhibitors**	TGF-β	?
**Cytokines regulated by activation of the HGF/Met axis**		
**Stimulated**	IL-11, IL-6, IL-8 VEGF, SCF, SDF-1α	
**Inhibited**	IFN-γ TNF-α, TGF-β	

Abbreviations: b-FGF—basic fibroblast growth factor; OSM—oncostatin M; HIF-1α—Hypoxia-inducible factor 1α; NF-κB—nuclear factor kappa B; TGF-β—tumour growth factor β; TNF-α—tumour necrosis factor α.

In CML, IL-3 was shown to promote the production of HGF at the mRNA and protein levels [16]. However, CD34+/CD38− stem cells and CD34+/CD38+ progenitor cells as well as chronic phase CML basophils expressed Met at low levels, and IL-3 stimulation did not induce Met expression on the surface of CD34+/CD38− stem cells or CML basophils.

Regarding MPNs, our group showed that levels of HGF, IL-11 and tissue inhibitor of metalloprotease 1 (TIMP-1) were high in the serum and bone marrow plasma of PV patients, and that HGF and IL-11 regulated each other’s production using paracrine and autocrinefeed-back loops involving BMSCs and glycophorin A+ (GPA+) erythroblasts (Figure 3B) [15]. An HGF/Met/IL-11/IL-6/gp130/STAT3 cascade was found to be activated in PV clonal erythroblasts (Figure 2B); activation of a similar autocrine HGF/IL-11/IL-6 cascade has been described in multiple myeloma and in solid tumours [94,115,116,117]. Consistent with HGF and IL-11 autocrine production in PV erythroblasts being independent from *JAK2*V617F, no correlation was found between *JAK2*V617F-mutated allele burden (% *JAK2*V617F) and HGF or IL-11 mRNA levels. Hoermann *et al.* recently reported that *JAK2*V617F induced OSM expression in malignant erythroid and megakaryocytic cells in a STAT5- and PI3K-dependent manner [118]. In turn, increased OSM expression induced HGF, VEGF and SDF-1 in bone marrow fibroblasts.

Additionally, Met expression is induced by IL-11, SCF or IL-3 on subsets of CD34+ hematopoietic progenitors (CD34+/CD33− and CD34+/CD38− cells) (Table 2) [91]. While the effect of IL-11 on Metis marginal, SCF clearly induces Met expression on early erythroid progenitors (BFU-E); SCF and HGF acted in synergy to promote the formation of BFU-E.

## 4. Activation of the HGF/Met Axis as an Early Event in MPNs

Growth factors are major regulators of haematopoiesis and contribute to haematopoetic stem cell (HSC) renewal, proliferation and differentiation, thus unbalanced cytokine expression could result in an abnormal haematopoiesis. The Bcr-Abl- and *JAK2*V617F-independent up-regulation of HGF production observed in malignant progenitors is expected to activate the HGF/Met pathways, in turn increasing the rate of progenitor proliferation and subsequently, the risk of somatic mutation occurrence in genes critical for myelopoiesis. Such genes would logically include genes encoding for myelopoietic cytokines, their receptors and other signalling molecules. These are precisely the kind ofgenes found mutated in CML and MPNs: Bcr-Abl; Mpl, receptor for Tpo; Jak2, main signalling molecule for myelopoietic cytokines; the role of calreticulin mutants remains to be clarified.

Moreover, several groups demonstrated that *JAK2*V617F is not the initial event in subsets of MPN patients: the *JAK2*V617F mutation can be preceded by other mutations or gene re-arrangements [54,61,119,120,121,122,123]. In addition, *JAK2* mutation or rearrangement was shown to occur several times in certain patients, which hints at strong pressure on Jak2 expression and function in MPNs. Thus, a model including an independent, possibly early deregulation of the HGF/Met axis would be consistent with the notion that *JAK2*V617F is not always the first event in MPNs.

## 5. Molecular Mechanisms of Activation of the HGF/Met Axis

There is very little information in the literature as to how the HGF/Met axis becomes activated in haematological malignancies, asides from evidence of overproduction of HGF [12,13,14,15,16,17,92,93,94,95,96,97,98,99,100,101,124]. Thus, this section summarizes the few studies that have attempted to explain the potential mechanisms of this deregulation.

### 5.1. Genetic Alteration of the HGF and MET Genes

Only one recent study identified a case of transactivation of HGF expression by fusion genes in AML patients with complex karyotypes [95]. Consequently, at this point there is no evidence of chromosomal alterations or mutationsthat may explain the widespread over-expression of HGF in myeloid malignancies. Several groups have searched for evidence of *MET* mutation, amplification or overexpression in myeloid malignancies. In 1994, Jücker *et al.* investigated METmRNA expression in myeloid cell lines (HL60, K562, U937) and did not detect any; in primary cells they detected MET mRNA for only 6/73 (8%) patients with haematological malignancy: 1 AML, 4 Hodgkin’s lymphoma, 1 Burkitt’s lymphoma (and none of 7 CML tested) [125]. The paucity of MET mRNA expression in myeloid malignancies may be explained in part by the frequency of partial or complete deletion of chromosome 7, where the *MET* and *HGF* genes are located. In UKE-1, a human cell line homozygous for the *JAK2*V617F mutation that lacks one chromosome 7, we found low levels of HGF mRNA and MET mRNA was not detectable. In contrast the human erythroleukaemic cell line HEL, also homozygous for the *JAK2*V617F mutation, showed very high HGF mRNA expression, and moderately high MET mRNA levels [15]. These observations are further evidence that the *JAK2*V617F status cannot explain overproduction of HGF in *JAK2*V617F-mutated MPNs. Of note, we sequenced the MET genes in the HEL and UKE-1 cell lines and found no mutation.

Recent studies of MPN genetics did not provide evidence of *MET* mutation in PV, ET or PMF [6,58,59,126]. Other studies looked at two *MET* variants, R970C and T992I (also referred to as R988C and T1010I) newly identified in solid tumours and haematological malignancies: AML, chronic myelomonocytic leukaemia (CMML), and chronic lymphocytic leukaemia (CLL). Both *MET* variants were present, jointly in most cases, in the cohorts tested (96 solid tumours, 191 AML, 96 CLL, 32 CMML; no CML and no MPN) but at very low frequencies (<2%) similar to those observed in healthy donors [127]. For six of the above patients, the *MET* variants were present in germline DNA. However, although these mutants are associated with hereditary papillary type I renal carcinoma, no transformative capacity could be found *in vitro* for the *MET*R970C and T992I variants. In the present high-throughput sequencing era, this emphasises the need to distinguish between truly oncogenic variants and rare single nucleotide polymorphisms (SNPs) with no functional consequence.

In summary, activating mutations of the *MET* gene are very rare in myeloid malignancies. Moreover, as long as capacity to cause disease onset or progression is not proven, some of the *MET* mutants discovered in patients may simply be variants [127]. Hence, it is unlikely that the very frequent activation of the HGF/Met axis observed in myeloid malignancies results from *MET* mutation. However, micro-re-arrangements of chromosome 7 involving the *MET* gene cannot be formally excluded.

### 5.2. MET Over-Expression

**Figure 4:**
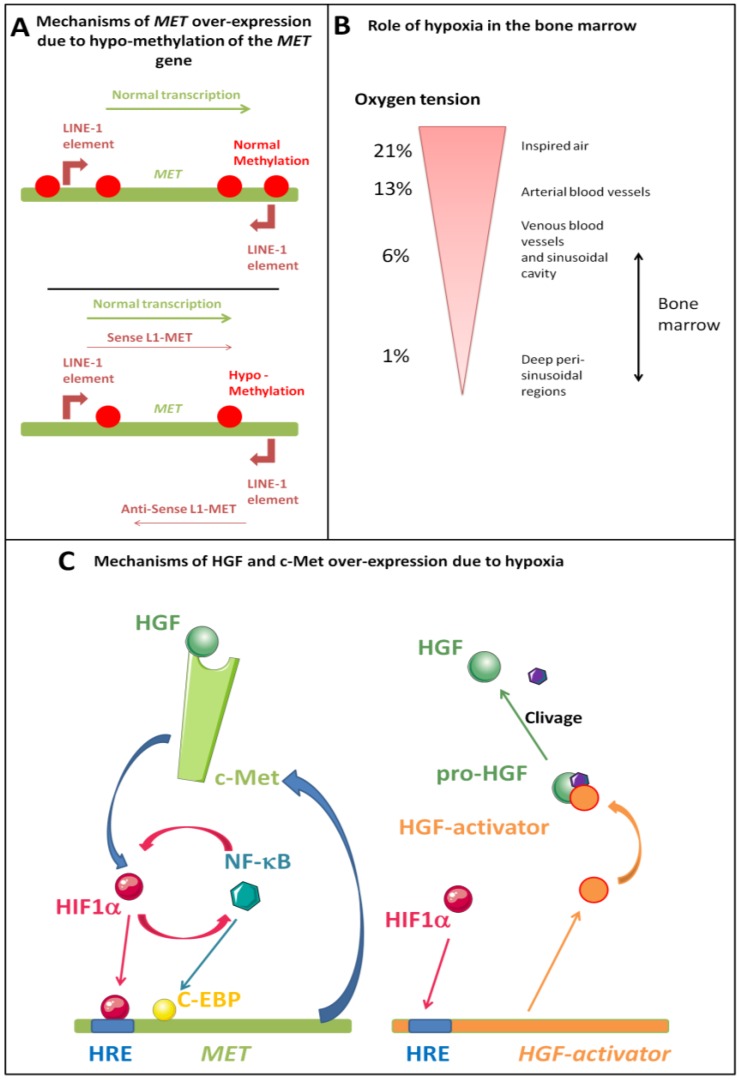
Molecular mechanisms of activation of the HGF/Met axis. (**A**) Mechanisms of MET over-expression: Hypomethylation of the LINE-1 (L1) retrotransposon promoter was reported during CML blast crisis; patients with hypomethylated L1 showed activation of MET transcription; (**B**) In chronic myeloproliferative diseases, cells accumulate in the bone marrow, which can result in local hypoxia and subsequent up-regulation of HGF. In mammals, air oxygen tension, around 21% when inspired, drops to around 6% in venous blood vessels and in the sinusoidal cavity of the bone marrow. In deep peri-sinusoidal regions, oxygen tension may be as low as 1%, indicating severe hypoxia in certain regions of the bone marrow; (**C**) HGF and Met expression can be induced by NF-κB and HIF-1α in a regulating loop, where NF-κB promotes HIF-1α, HGF and Met expression, and HGF may in turn regulate NF-κB negatively. The HGF activator (HGFA), a serine protease that cleaves pro-HGF into active HGF, is also regulated by hypoxia.

In cancer HGF is produced by malignant cells and by surrounding fibroblasts, and thus HGF acts on Met in an autocrine and paracrine manner. In solid tumours, the most frequent scenario responsible for the activation of the HGF/Met axis is overexpression of the Met receptor in absence of mutation [128,129]. One hypothesis is that increasing expression of Met facilitates oligomerisation and subsequent activation in a ligand-independent fashion. In CML, hypomethylation of the LINE-1 (L1) retrotransposon promoter was reported to be more frequent in blast crisis, compared to chronic phase, and patients with hypomethylated L1 showed activation of MET transcription (Figure 4A) [129]. *MET* overexpression was found in 61% of CML with L1 hypomethylation, while MET mRNA was not expressed at all in CML with methylated L1. Consistently, Met was expressed at the surface of CD34+ progenitors and granulocytes of L1 hypomethylated CML patients. Differences in *MET* expression were not due to *MET* endogenous promoter, which was found unmethylated both in CML patients and in healthy individuals. Of note, L1 hypomethylation (Met expression) was associated with poorer cytogenetic response of CML patients to interferon or imatinib, and poorer progression-free survival [129]. Regarding MPNs, our group found that MET mRNA levels were significantly higher in PV erythroblasts (median: 234 MET mRNAcopies/1000 RPLP0 mRNA copies) than in controls with secondary erythrocytosis (median: 77 MET mRNAcopies/1000 RPLP0 mRNA copies) [15]. To our knowledge, MET expression has not been studied in ET and PMF.

### 5.3. HGF Over-Expression

#### 5.3.1. Role of Bcr-Abl and the Jak2V617F, MplW515 or CalR Mutants

Various events happening during disease progression may result in activation of the HGF/Met axis. In murine FL5.12 cells, retroviral transformation by Bcr-Abl induced VEGF, FGF-2, HGF, IL-8, MMP2 and MMP9, but HGF production was Bcr-Abl-independent in CML, as it could not be blocked by imatinib [16,97]. Interestingly, Bcr-Abl-positive CD34+ cells from CML patients show significant reduction in telomere length, which was correlated with disease progression [130,131]. Such cells present a particular “telomere-associated secretory phenotype” profile that includes interleukins and other moleculesthat potentially drive progression to accelerated phase; over-expression of Met and pro-MMP9 is part of this profile [132]. Altogether it appears that expression levels of both HGF and Met do not directly depend on Bcr-Abl in CML.

Regarding MPNs, it is established that IL-6 and OSM can be induced by the *JAK2*V617F mutation in a STAT5 and PI3K dependent manner [15,118]. In turn, OSM induces the expression of HGF, VEGF and SDF-1, notably by bone marrow fibroblasts (Table 2). Logically, the production of OSM induced by *JAK2*V617F, and subsequentOSM-induced HGF increase, were abrogated in a dose dependent manner by Jak inhibitors. In this context, activation of HGF production was an indirect consequence of the deregulation of another cytokine by *JAK2*V617F. However, we did not observe any significant increase of OSM levels in PV patients, and we showed that PV erythroblasts produce HGF and IL-11 in excess and independently from the *JAK2*V617F mutation [15]. This of course does not exclude some degree of involvement of OSM in the increased levels of HGF in subsets of PV patients, or in other MPNs. Overexpression of HGF in PV clonal cells was also observed by Berkofsky-Fessler *et al.*, who used Affymetrix arrays to study gene expression in PV and normal CD34+ progenitors [133].

The recent report that myeloid progenitors transformed with the proto-oncogene SKI (v-ski or avian sarcoma viral oncogene homolog) later become partially dependent on HGF signalling, also suggests the possible occurrence of independent (passenger) events causing overexpression of HGF, secondary to transformation but not directly dependent from the main oncogenic event [134]. Altogether, the literature and our own studies support an indirect stimulation of the HGF/Met axis in chronic myeloid malignancies, by other cytokinesorby additional oncogenic events occurredeither in the early stages of transformation or/and during disease progression.

#### 5.3.2. Role of Hypoxia

One of the hallmarks of chronic myeloproliferative diseases and AMLs is ahypercellular bone marrow, whichcreates a hypoxic environment. Very recently, Spencer *et al.* measured the absolute oxygen tension in the bone marrow of live mice using two-photon phosphorescence lifetime microscopy. Despite a high vascular density, they found low oxygen concentration with a lowest oxygen concentration (around 1%) in peri-sinusoidal regions (Figure 4B) [135]. In their 2006 review, Boccaccio *et al.* described in detail the involvement of the hypoxia-responsive pathways in the promotion of stem cell growth and in the regulation of Met expression [128]. Met is up-regulated by HIF-1, the oxygen sensor, and the *MET* promoter contains several putative hypoxia-response elements (HREs). Two of the HREs were found to be functionally active, and they are located after the transcription start site alongside an AP-1 siteknown to be involved in MET transcription in response to hypoxia (Figure 4C). Previously, Tacchini *et al.* had shown, in the hepatocyte cell line HepG2that HGF increased the expression of the α subunit of HIF-1 (HIF-1α) via the PI3K pathway thus augmenting HIF-1α DNA binding activity (and in turn, MET expression) [136]. In addition, HGF treatment increased the expression of HIF-1α target genes, heme oxygenase, urokinase plasminogen activator (uPA) and uPA receptor (uPAR), a receptor also used by TIMP-1, a negative regulator of HGF signalling. The HGF activator (HGFA), a serine protease that cleaves pro-HGF into active HGF, is also regulated by hypoxia; the promoter of HGFA contains two putative HREs [137]. In pancreatic cancer cells hypoxia increased the expression of HGFA mRNA and protein, and this effect was inhibited by a siRNA targeting HIF-1α. This hypoxia-mediated regulation of HGFA also occurs in haematopoietic cells, notably the monocytic and B-lymphoid lineages.

Thus, the hypoxic environment generated by bone marrow hypercellularity promotes the up-regulation of HGFA, HGF and Met expression. In turn, activation of the HGF/Met axis stimulates the expression of HIF-1αin a positive feedback loop that maintains HGF/Met activation. Interestingly, CML progenitors seem to be more dependent onHIF-1αsignalling than normal progenitors for their survival. When HIF-1α^−/−^ CML cells were introduced in recipient mice, repopulation by HIF-1α^−/−^ cells was dramatically reduced compared to wild type, HIF-1α^+/+^ CML cells [138].

#### 5.3.3. Role of NF-kB

The transcription factor NF-κB controls the expression of many inflammatory cytokines [139]. Under hypoxic conditions, NF-κB is induced via HIF-1α-independent mechanisms (Figure 4C) [140]. NF-κB modulates the basal expression of HIF-1α mRNA and its normoxic protein level [141]. Met expression can be induced by NF-κB and c/EBP, via TNF-α stimulation [142]. Inversely, in case of HGF stimulation, NF-κB inactivation was shown to depend partially on GSK3β phosphorylation [143]. These observations suggest that a regulating loop exists, whereNF-κB promotes HIF-1α, HGF and Met expression, and HGF may in turn regulate NF-κB negatively (Table 2).

## 6. HGF Overproduction as Prognosis Marker of Disease Severity

The role played by HGF in the maintenance of stem cells and in the survival, proliferation and migration potential of malignant cells, suggested that HGF expression levels could have independent prognostic value in myeloid malignancies. Thus, it was not surprising to find accumulating evidence linking HGF levels to biological parameters such as white blood cell counts and microvessel density of the bone marrow. An early study by Hino *et al.* showed reducedHGF levels in the bone marrow plasma of AML patients in complete remission (CR) [92]. Hjorth-Hansen *et al.* found that HGF levels measured in serum from 60 AML patients at the time of diagnosis were correlated with peripheral blood blast counts, disseminated intravascular coagulation, and lysozyme (reflecting monocytic differentiation and tumour burden) [124]. In agreement with Hino *et al.*, AML patients in CR also had undetectable HGF. Later Verstovsek *et al.* found the level of HGF in AML and MDS patients correlated with white blood cell and monocyte counts; high levels of HGF were also associated with shorter survival in AML, but not in MDS [96]. In a multivariate analysis, HGF was found to be a significant, independent prognostic factor in AML. In 2005, Kim *et al.* investigated the association between HGF levels in AML and CML and clinical parameters [17]. Serum levels of HGF were also significantly increased in AML and correlated with white blood cell counts and serum levels of lactate dehydrogenase (LDH). In univariate analysis, HGF and age were significant predictors of achievement of CR in AML but in multivariate regression analysis, HGF was the sole significant predictor of CR. Again, the leukaemia-free survival rate of patients with low HGF levels was better than that of AML patients with high HGF levels.

In CML, several studies found that serum levels of VEGF and HGF were significantly increased and correlated with white blood cell counts and serum LDH levels; one group concluded that high blood levels of HGF correlated with poor prognosis and survival [16,17].

Similarly, high HGF levels in serum were also associated with poor prognosis in myeloma: serum HGF levels decreased when the treatment proved effective, and increased again towards relapse [78]. In the late 90s the Nordic Myeloma Study group concluded that high serum HGF levels could be used to identify patients with a poor response to treatment, suggesting that the HGF level reflects tumour burden and could be a marker of disease activity [17,144,145].

Hence the serum HGF levelhas been proposed as an independent prognostic marker in myeloma, in AML, andin CML but in MPNs, such studies have not yet been performed in large cohorts of patients. We found that the *JAK2*V617F mutation was neither required nor sufficient to induce HGF production but PV patients presenting at the time of diagnosis with a high *JAK2*V617F allelic burden also had high HGF levels [15]. HGF levels were correlated with blood neutrophil counts, a finding consistent with HGF level being the reflection of tumour burden. Tefferi *et al.* later analysed a cohort of 127 patients with PMF and again, the level of HGF was found to be correlated with marked splenomegaly and leukocytosis [99]. Another study from the same group confirmed that the level of HGF was correlated with leucocytosis in PV; however, multivariate and univariate analysis failed to show a correlation between HGF level and prognosis in PV [100].

Altogether, in AML and possibly in CML, the level of HGF in serum strongly correlates with biological parameters considered to reflect tumoural burden, especially leukocyte counts, splenomegaly and vascular syndrome. However, the value of HGF as prognostic marker of disease progression or patient survival is not established in MPNs and remains uncertain in CML. The literature shows conflicting results, possibly due to differences in study design (e.g., source of HGF-serum *vs.* plasma, blood *vs* bone marrow; analysis at the time of diagnosis or during treatment; small size of the patient cohorts in some studies, *etc.*). Thus, the analysis of large cohorts of patients is necessary to conclude decisively on the predictive value of HGF levels in CML and in MPNs.

## 7. The HGF/Met Axis as a New Target in the Treatment of CML and MPNs

Since HGF levels reflect tumour burden and activation of the HGF/Met signalling pathways plays a key role in carcinogenesis, it was logical that the pharmaceutical industry developed therapeutic strategies aiming at inhibiting the HGF/Met axis. Such therapeutic approaches encompass direct inhibition of HGF, or blocking its binding to Met, or neutralising antibodies targeting HGF or Met, and small molecule inhibitors, used as single agents or in combination with other drugs specifically targeting the main biological marker(s) of the malignancy of interest (in CML: Bcr-Abl; in MPNs: *JAK2*V617F).

A growing body of evidence demonstrates the efficacy of HGF and Met inhibitors, alone or in combination therapy, in solid tumours of various types and in myeloma. In contrast, there are no published studies of pre-clinical or clinical use of HGF or Met inhibitors in CML or MPNs. One likely reason isthat CML and MPNs are considered to be the consequence of a single genetic marker; consequently the drugs tested in these diseases primarily target Bcr-abl (CML) and Jak2 (MPNs). However, CML patients develop resistance to Bcr-Abl inhibitors, Jak inhibitors have little or no effect on the *JAK2*V617F burden, and so far no CML or MPN patient has been cured. Given the experience and success of combination therapies in solid tumours, and similar chronic inflammation in cancer and in MPNs, one can safely postulate that the combination therapies used in solid tumours will in time be adapted to MPNs. Meanwhile, combination treatments currently tested in patients with solid tumours or myelomashould establish whether blocking the activation of the HGF/Met pathways is truly useful.

A plethora of Met inhibitors have been developed; our aim in this review is not to provide an exhaustive list, nor the detailed mechanism of action of each molecule, available for instance on the links [146,147] for the on-going clinical trials, and in reviews [148,149]. Rather, we will present the main categories of molecules capable of blocking the HGF/Met axis, according to their mode of action.

### 7.1. Abl-Bcr Inhibitors

*In vitro* imatinib enhanced HGF/Met-induced motility response of thyroid cancer cells, whereas c-Abl was shown to be required for Met-dependent oncogenic transformation in the GTL-16 and HepG2 cell lines [150]. Imatinib and nilotinib treatment resulted in reduced proliferation and survival of the two cell lines, via the p38 MAPK/p53 pathways [151]. In practice, at Food and Drug Administration (FDA)-approved doses, imatinib does not inhibit Met kinase activity.

### 7.2. Neutralising Antibodies

HGF and Met neutralising antibodies are best suited to diseases that depend on paracrine/autocrine HGF, the most frequent mechanism of activation of the HGF/Met axis in MPNs. *In vitro* Met neutralising antibodies were found to block efficiently the growth of *JAK2*V617F-mutated cells, including PV erythroblasts and the HEL cell line, which expresses HGF at very high levels [15]. Burgess *et al.* reported a panel of five anti-HGF antibodies able to inhibit tumour growth in an autocrine HGF/MET-driven xenograft model of glioblastoma [152]. These antibodies acted by preventing the binding of HGF to Met, thus impairing the activation of downstream signalling. AMG102 (rilotumumab), a fully humanised anti-HGF antibody and one of the most advanced antibody in clinical development, has been in phase III trial since 2012. L2G7, another anti-HGF antibody successfully inhibited tumours of the central nervous system in an intracranial xenograft model. Anti-Met antibodies directed against the extracellular domain of the receptor have been developed as monovalent antibodies after it was observed that bivalent antibodies could generate an agonist effect. One-armed variants of the Met antibody 5D5, CE-355621 and DN-30 showed promising activity in various solid tumours [20]. DN-30 was shown to inhibit gastric cancer in a xenograft model by stimulating Met shedding [153]. The LMH-87 antibody was shown to inhibit tumour growth of U87 glioblastoma cells in a xenograft model [153]. A bivalent antagonist antibody called 11E1 has also been described but its mechanism of action is still unclear. Currently several antibodies targeting Met are available for use in humans in the context of clinical trials; MetMab (onartuzumab), a monovalent antibody that competes for the binding of HGF, is one of them [20].

Recently, the novel concept of nanobodies was introduced for therapeutic use in HGF/Met therapy [154]. Nanobodies are small (~15 kDa) therapeutic proteins. They are the smallest functional fragment of heavy-chain antibodies, and are naturally occurring in Camelidae. They retain the full antigen-binding capacity of the original antibody and can be associated with other nanobodies, molecules, protein domains or drugs. The anti-Met nanobody was tested in an *in vitro* myeloma model, where it showed high specificity and efficiently blocked HGF/Met signalling, resulting in decreased cell proliferation [154].

### 7.3. MET Small Molecule Inhibitors

Small tyrosine kinase inhibitors are now approved therapies by the FDA (imatinib, erlotinib, lapatinib, sunitinib, sorafenib). Small molecule inhibitors can be efficient in the context of ligand-dependent or ligand-independent activation. There are two major types of small molecule Met inhibitors. The ATP dependent inhibitors are based on the structure of the ATP binding pocket of the receptor tyrosine kinase (RTK). As this is a well conserved structure amongst RTKs, they tend to have overlapping activities and also inhibit other RTKs, but with different affinities. The non-ATP dependent inhibitors rely on less conserved trans-activation or dimerization sites [155].

The MET-related RON receptor is a frequent secondary target of Met inhibitors [156]. Ron is expressed in normal CD34+ haematopoietic cells and its tyrosine kinase domain shares 80% identity with that of Met, making RON a likely target for inhibition by molecules directed against this common region. Like HGF, RON regulates cell proliferation, migration and survival. The short form of RON (sfRON), which lacks the extracellular domain, is not found on normal CD34+ cells but is present on the surface of AML CD34+ cells. Small molecule Met inhibitors SU-11274 and PHA-665752 decreased the survival of sfRON-expressing AML cells in a dose dependent manner. Hence, it is worth keeping in mind that sfRON might show similar downstream biological effect as Met; that they are both deregulated in AML and can be targeted efficiently by the same inhibitors targeting shared regions of their kinase domain.

Other small molecule inhibitors have been developed. NK4 is an internal fragment of HGF that is composed of the NH2-terminal domain and 4-kringle domains. It binds to Met without triggering its activation, thus preventing downstream signalling. NK4 also inhibits angiogenesis induced by VEGF and b-FGF. NK4 has been shown to efficiently inhibit angiogenesis, tumour growth and tumour metastases in colorectal and pancreatic cancers *in vivo*. Met inhibitors SU11274 and PHA-665752, which canblock the response to HGF in a myeloma model, have been studied in preclinical models [157]. One concern is that MET mutated variants could show an altered sensitivity to the inhibitors; the possibility of MET variants leading to resistance to Met inhibitors was also raised in solid tumours and in CML [79]. Depending on where the inhibitor binds the receptor, the variants present in that region of the receptor might impair the affinity of the inhibitor and then its ability to efficiently block MET activation.

Inhibitors of HGF activators (HGFA), notably anti-HGFA antibodies aiming at preventing HGFA to cleave pro-HGF into its active form, are also being developed [20].

Met inhibitorsPF-02341066 (crizotinib) and SU-11274 both suppress the growth of basophil-lineage committed KU812 and uncommitted K562 cell lines in a dose dependent manner. They also inhibit the proliferation of CML bone marrow and peripheral blood mononuclear cells [16]. Recent studies demonstrated that concomitant inhibition of FGFR1 and MET (crizotinib) blocked the activation of HGF and resulted in sustained cell killing both *in vitro* and *in vivo* in a xenograft model of FOP2-FGFR1 aggressive myeloproliferative syndrome/leukaemia [95]. SU11274 inhibits colony formation and reduces viability in A9M, U937 and OCI-AML cells. In U937 and OCI-AML cells treated with SU11274, a differentiation effect was also observed with treated cells appearing less blast-like and more differentiated [158].

MP470 (amuvatinib), a multipotent kinase inhibitor, was shown to have inhibitory effects on the HGF/MET signalling pathways in an *in vitro* model of myeloma [159]. Another study on myeloma cell lines showed that targeting Met with SU11214 is relevant for the treatment of resistant cells characterized by increased Met signalling activity [160]. In the cytokine-independent Ba/F3-TprMet model, SU11214 induced G1 cell cycle arrest and apoptosis via the activation of caspase 3. Interestingly, SU11214 also blocked the Ras, PI3K/Akt, Gsk3β pathways [160]. Recently, Tiedt *et al.* conduct a study to predict resistance to Met inhibitors using the murine Ba/F3-Met expression cell line [79]. Exposure to NVP-BVU972 or AMG458 induced point mutations in the Met transgene, preferentially in position F1200. They characterized several of the mutants and showed that NVP-BVU972 had no impact on the phosphorylation of mutant Met compared to wild type Met, and that AMG458 had impact only on some of the mutants [79]. Also, IL-6-induced HGF transcription and production, which in turn stimulates IL-10, an anti-inflammatory cytokine, was reversible with the use of the SU11274 Met inhibitor [113].

### 7.4. Interferon (IFN)-α

**Figure 5:**
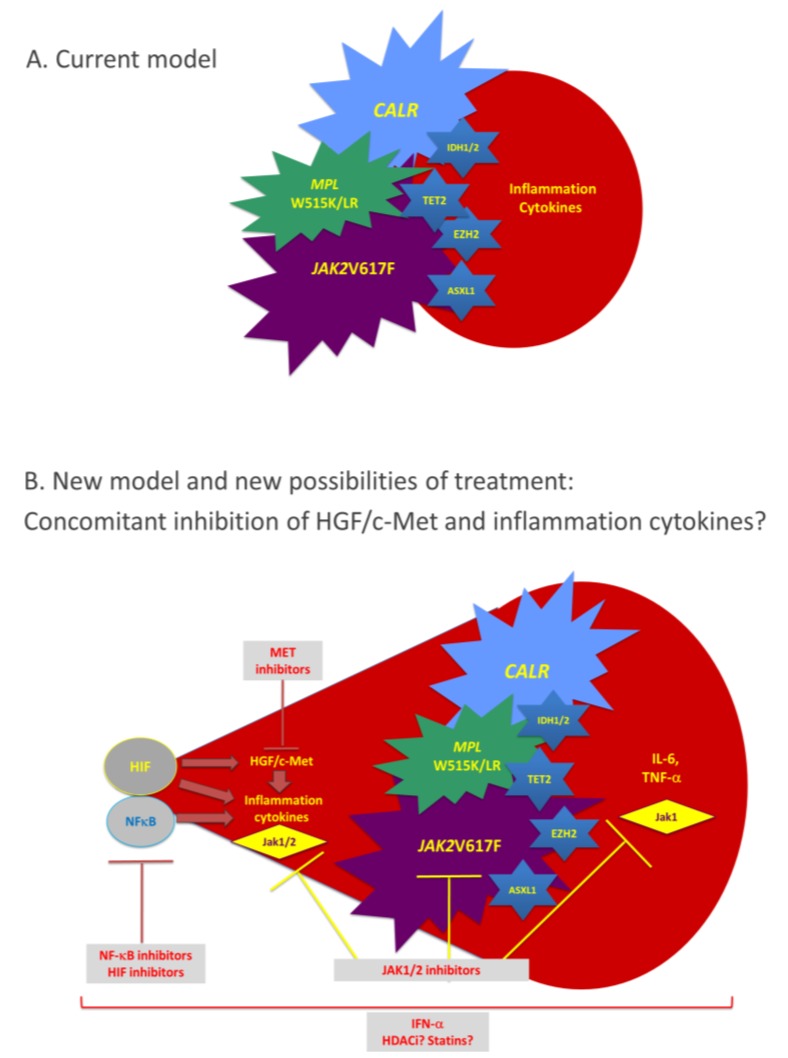
The HGF/Met axis as a new target in the treatment of MPNs. (**A**) The current pathogenic model for MPNs states that that disease is initiated by one the three main markers: the *JAK2*V617F mutation, *CALR* or *MPL*W515L/K mutants. The mutants result in constitutive activation of the Jak2/Stat5 pathways, and the increased production of inflammation cytokines typically found in MPNs is considered to be a consequence of the *JAK2*, *CALR* or *MPL* mutants. Additional, passenger genetic events, such as mutation in the *EZH2*, *ID1/2*, *TET2* or *ASXL1* genes, may occur and contribute to a more severe disease evolution; (**B**) The new model takes into account the *JAK2*V617F-independent contribution of HGF/Met to the pathogenesis of MPNs. This model is consistent with the demonstration by several groups that *JAK2*V617F is not sufficient to sustain MPN disease, and that *JAK2*V617F is not the first event in subsets of patients. In the new model, activation of the HGF/Met axis due to bone marrow hypoxia (HIF-mediated activation) or/and exposure to inflammation cytokines (NF-κB-mediated activation), lead to enhanced survival and proliferation of mutated myeloid progenitors and increased production of various cytokines responsible for inflammation, neo-angiogenesis, and fibrosis. Therefore, efficient therapy for MPNs should aim to block simultaneously the cascade of inflammation cytokines, their signalling and the main disease marker (*JAK2*, *MPL* or *CALR*). This may be achieved by combining JAK1/2 inhibitors and molecules efficient at blocking NF-*k*B- and HIF-mediated activation of the HGF/Met pathways.

Several groups have shown that IFN α-2a is efficient in the treatment of MPNs; in fact, IFN α-2a remains the only molecule capable of inducing complete clinical, biological and *JAK2*V617F molecular remission in PV [161,162]. In this regard, it is interesting to note that IFN-α decreases the production of many inflammatory cytokines and inhibits or suppresses the expression of HGF and Met (Table 2) [163]. For instance, CML patients treated with IFN-α were reported to present less HGF than those treated with HU or untreated CML patients [164]. The remarkable efficacy of IFN α-2a in myeloproliferative diseases is likely due to the inhibition of a broad range of cytokines and receptors implicated in myelopoiesis and inflammation (Figure 5). However, IFN-α has side effects that lead certain patients to discontinue treatment. Using IFN-α in combination with other molecules would allow to reducing IFN-α doses and side effects, while potentially increasing the chance of curative treatment.

### 7.5. Other Molecules

As described above, HGF and Met expression depend on NF-κB and HIF-1α, and HGF production can be induced by IL-3 and OSM, thus indirectly by the activation of Jak1/Jak2 (mostly Jak1) signalling. This suggests that inhibitors of NF-κB, HIF-1α and of course JAK1/2, could be useful by reducing inflammation symptoms and the survival of clonal cells, independently from the presence of the *JAK2*V617Fmutation(s) (Figure 5). Fortunately numerous HIF-1αor NF-κB inhibitors already exist and several are being tested in clinical trials [165,166,167,168,169,170,171,172,173,174]. JAK1/2 inhibitors will not be discussed here since they are already used in the treatment of PMF and severe forms of PV, initially with the aim to block *JAK2*V617F, then to reduce severe inflammation-linked symptoms. Moreover, JAK1/2 inhibitors used as single agents are not capable of reducing the size of the *JAK2*-mutated clone in MPN patients.

In solid tumours, increased tumoural HIF-1α expression has been correlated with increased aggressive disease and poor patient prognosis. Consequently HIF-1 has become an interesting new target in cancer treatment. Numerous small molecule HIF-1 inhibitors havebeen identified, and a few are in clinical trials. The different types of HIF-1 inhibitors, their mode of action and their efficacy in clinical trials, mostly in the context of solid tumours and myeloma, have recently been reviewed [165,166]. In addition, existing anti-cancer drugs, although not targeted at HIF-1, are known to act in part by inhibiting HIF-1 activity: these drugs include PI3K/Akt/mTOR inhibitors, Hsp90 inhibitors, histone deacetylase (HDAC) inhibitors, proteasome inhibitors (bortezomib), as well as several natural compounds such as berberine, an alkaloid used in traditional Chinese herbal medicine, or curcumin [167,168,169,170,171]. HDAC inhibitors or bortezomib, PI3K/Akt/mTOR inhibitors, and Hsp90 inhibitors, have been or are being tested in CML and MPN patients [169,170,171,172]. However, so far these drugs have mostly been tested as single agents; combination treatments would be more likely to be successful [168,169]. Moreover, in MPNs HIF-1 may not be the best HIF target, asHIF-3 mRNA was found expressed at higher levels than HIF-1 in PMF. In contrast to HIF-1, HIF-3 does not induce Epo, an interesting characteristic since low Epo is a hallmark of MPNs [172]. Thusfor the treatment of MPNs, it may be best to select HIF inhibitors on their capacity to inhibitthe activity of HIF-3, not HIF-1.

The anti-apoptotic cytoprotective effect of NF-κB implied that it could be a useful therapeutic target for the treatment of hematologic malignancies. Indeed, several drugs with proven efficacy for the treatment of myeloma, such as proteasome inhibitors, thalidomide, lenalidomide and arsenic trioxide, block NF-κB activation. Triptolide (diterpenoid triepoxyde), a purified component of a traditional Chinese medicine, inhibits the expression of NF-κB and various NF-κB-regulated genes. NF-κB activation can be also inhibited by IKKβ-selective inhibitors [173]. In CML cell lines and primary CML cells from patients, pristimerin induced apoptosis in imatinib-resistant cells harbouring the T315I mutation by blocking NF-kappaB signalling as well as the expression of NF-kappaB-regulated genes [174]. Hence, pristimerin could be useful in CML to overcome imatinib resistance. The proteasome inhibitor bortezomib was tested in a murine model of myelofibrosis induced by thrombopoietin (THPO) overexpression. Bortezomib was able to inhibit Tpo-induced NF-κB activation *in vitro* in murine megakaryocytes and *in vivo* in THPO(high) mice, and dramatically improved THPO (high) mouse survival (89% *vs.* 8% at week 52) [175]. Consequently, bortezomib was tested as a single agent in patients with advanced stage PMF or post-PV/post-ET myelofibrosis: neither remission nor clinical improvement was recorded in 16 patients [171]. Again, single agent therapy is unlikely to be successful in MPNs, especially for patients in advanced stages.

More recently, the combination of Ruxolitinib (RUX), a Jak1/2 inhibitor, and Panobinostat (PAN), a pan-deacetylase inhibitor (pan-DACi), has been tested in a dose-finding phase 1B study in patients with PMF or with myelofibrosis secondary to PV or ET [176]. The combination of RUX and PAN demonstrated a tolerable safety profile with encouraging efficacy as demonstrated by spleen responses in patients with intermediate- and high-risk myelofibrosis.

## 8. Combination of Inhibitors of the HGF/Met Axis and Drugs Commonly Used in the Treatment of MPNs or CML

Human malignancies are very rarely driven by a single mutant and as a result, inhibiting a single target is unlikely to be sufficient if one has curative treatment in view; combination therapies are much more likely to be effective. In solid tumours and in myeloma, Met inhibitors have already been tested in combination with other signal transduction inhibitors, and some combinations have proven to have synergistic action [21,177,178]. For instance the EGFR inhibitor gefitinib has been tested jointly with the HGF inhibitor NK4 in a preclinical model of gastric cancer, and this combination has proved more efficient than either agent on itsown [179]. Met inhibitors have also been combined with chemotherapy in models of solid tumours. The anti-HGF antibody AMG102 has been used with temozolomide or docetaxel *in vitro* and *in vivo* in glioblastoma models, and a synergistic effect on tumour growth was observed. Similarly, SU11274was able to reverse the resistance to cisplatin *in vitro* in U87H glioblastoma cells, and NK4 showed *in vivo* anti-tumour activity when associated with either cisplatin or gemtacibin in gastric and pancreatic models.

The combination of imatinib and Met inhibitors has been tested. In imatinib-treated tumours, levels of Met and phospho-Met were increased. Combination of PF2341066 and imatinib reduced Met and phospho-Met levels and suppressed metastasis [180]. In CML, imatinib and nilotinib were shown to induce the secretion of GM-CSF, a cytokine that mediates cell resistance via activation of the Jak2/Stat5 pathways [181]. Interestingly, GM-CSF is over-expressed in imatinib-resistant CML patients, compared to imatinib-sensitive CML patients in chronic phase [182]. Disruption of Jak2/Stat5 activity with AG490, a Jak2 inhibitor, suppressed the GM-CSF-mediated resistance, which suggests that JAK inhibitors could help eradicate imatinib- or nilotinib- resistant cells in CML.

Tétreault *et al.* analysed the effects of HGF and hydroxyurea (HU) in human gastric epithelial (HGE) cell lines [183]. HU inhibits DNA replication and is frequently used in the treatment of MPNs. In the presence of 20 mM HU, they demonstrated cytokine-independent cell proliferation. When HGF (3–20 ng/mL) was added, HU still inhibited cell proliferation although no significant inhibition of the HGF/Met pathways could be detected. These results suggest that combining HU with Met inhibitorsis possible, and could be a good, synergistic combination for the treatmentof MPNs.

**Figure 6:**
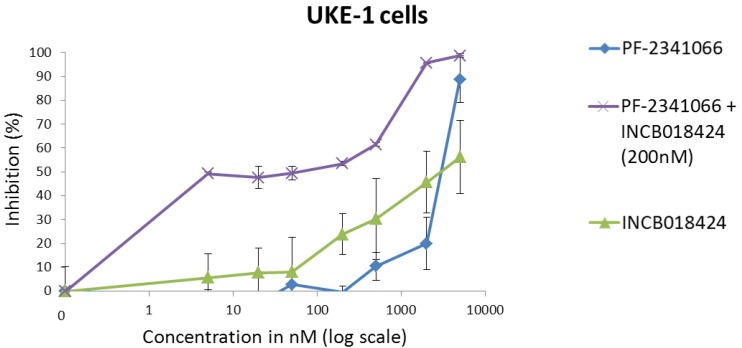
Synergy of MET and JAK inhibitors in *JAK2*V617F-mutated cells. MET/ALK inhibitor PF-2341066 was tested alone and associated with anti-JAK1/2 inhibitor INCB018424 on the *JAK2*V617F^+/+^ UKE-1 cell line. Only a weak inhibition was observed (at ≥1 μM) when molecules were tested separately. When UKE-1 cells were treated simultaneously with INCB018424 (200 nM) and PF-2341066 (increasing doses), synergistic growth inhibition (>50%) was obtained with 5 nM PF-2341066.

Our group tested the efficacy of combining MET and JAK inhibitors on the proliferation of the *JAK2*V167F homozygous HEL and UKE-1 cell lines. In both cell lines, only a weak inhibition was observed when molecules were tested separately, even at high doses. In contrast, more than 50% growth inhibition was obtained in UKE-1 cells treated simultaneously with reduced doses of PF-2341066 and INCB018424 (Figure 6). However, the same combination had no significant inhibiting effect in the HEL cell line, which produces HGF at very high levels.

Hence, it is feasible to design therapeutic protocols combining drugs that target cytokines at different levels, *i.e.*, production (NF-κB inhibitors, HIF inhibitors, IFN-α, HDAC inhibitors), receptors (Met inhibitors) or signalling pathways (JAK inhibitors, Bcr-Abl inhibitors), should allow to eradicate MPN clonal cells. Since each patient potentially differs in term of HGF production, inflammation cytokines, Met and Jak1/2 expression, as well as driving or/and passenger mutations potentially able to alter response to cytokines or/and treatment, efficient and eventually, curative combination therapies in MPNs will have to be personalised, adapted to the patient’s cytokine and mutation profile. Fortunately, the technology (cytokine profiling, next-generation sequencing) and many of the drugs are already available.

## 9. Conclusions

As in other malignancies, the HGF/Met axis is activated in CML and in MPN progenitors, due to the overproduction of HGF in the presence of normal or increased expression of Met. The cells that produce HGF include stromal cells and malignant cells: basophils in CML, clonal myeloid progenitors in MPNs, *JAK2*V617F-mutated or not. Studies in larger cohorts of MPN patients are needed to validate that the HGF level in serum reflects the tumour burden, and thus could be used as a prognostic marker. The mechanisms responsible for the overexpression of HGF are not fully understood but they appear to involve NF-κB, cytokine stimulation, and hypoxia (HIF-1 or/and HIF-3). In contrast to certain inflammation cytokines (IL-6, TNF-α), which depend on Bcr-Abl or *JAK2*V617F (Figure 5A), the autocrine production of HGF is independent from Bcr-Abl in CML, and independent from *JAK2*V617F in MPNs. Hence, deregulation of the HGF/Met axis could be either an early or a passenger event in MPNs, which significantly changes the pathogenic model of MPNs (Figure 5B). The new MPN model including the dependence factor HGF, which promotes cell survival and proliferation, is consistent with the recent evidence that the *JAK2*V617F mutation, even in the homozygous state, is not sufficient to sustain MPN disease [184,185]. This opens numerous new opportunities of treatment, since blocking HGF/Met function can be achieved with various types of inhibitors acting at different levels: gene expression, cytokine production, signalling (Figure 5C). New therapeutic protocols based on the expected synergistic action of molecules capable of blocking activation the HGF/Met axis, used in combination withBcr-Abl inhibitors (CML) or JAK inhibitors (MPNs), can now be envisioned. Importantly, these new therapeutic approaches may allow the preventing acquisition of drug-resistance, and lead to curing subsets of MPN patients.
